# Targeting ADAMTS-7: a vaccination against atherosclerosis – and its complications?

**DOI:** 10.1161/CIRCULATIONAHA.122.063495

**Published:** 2023-02-27

**Authors:** Thorsten Kessler, Heribert Schunkert

**Affiliations:** 1 German Heart Centre Munich, Department of Cardiology, Technical University of Munich, 80636 Munich, Germany; 2 German Centre for Cardiovascular Research (DZHK e.V.), partner site Munich Heart Alliance, 80636 Munich, Germany

Multifactorial diseases require multifactorial treatment. A good example is heart failure, for which – over the years – escalation to four (or more) different drug classes markedly improved outcome. In contrast, the progress in preventive treatment of atherosclerosis is characterized by a stepwise intensification of lipid lowering therapy. Other modalities such as antiplatelet or anti-inflammatory agents are preferentially recommended in advanced stages of disease and have yet to demonstrate a positive benefit/risk ratio in primary prevention. Thus, the search for therapeutic targets for preventing atherosclerosis is still in its infancy. Obviously, the hundreds of gene variants linked by high-throughput studies to the pathogenesis of atherosclerosis^1^ should reveal more targets for therapeutic modulation.

From a historical point of view, the most successful approach to eradicate a disease has been vaccination. Regarding atherosclerosis, some preclinical evidence is available for vaccines targeting lipid metabolism or the immune system^2,3^. In this issue of *Circulation*, Ma and colleagues use a different target class and pursue the approach of vaccination targeting ADAMTS-7^4^, an extracellular matrix protease that was among the first risk genes to be identified by genome-wide association studies (GWAS) for coronary artery disease (CAD). In fact, *ADAMTS7* resides at one of the most robustly replicated CAD risk loci^5,6^. The elucidation of its function in vascular biology and disease, however, is still in its early stages. Experimental work pointed to a role in modulating vascular smooth muscle cell (VSMC) function. Via degradation of its *bona fide* substrate cartilage oligomeric matrix protein (COMP), ADAMTS-7 was shown to promote VSMC migration and, thereby, neointima formation^7^. This phenotype, however, also relies on effects on endothelial cell migration and proliferation via degradation of another ADAMTS-7 target: thrombospondin-1^8^. In addition to this influence on vascular remodeling, experimental studies revealed that a lack of ADAMTS-7 or its catalytic activity reduce the burden of atherosclerosis in mouse models^9,10^.

Ma and colleagues now generated a vaccine targeting ADAMTS-7 using three epitopes of its secondary structure at the catalytic domain as well as the C-terminal thrombospondin (TS)-repeats^4^. This is important as the catalytic domain was recently identified to be critical for ADAMTS-7’s role in atherosclerosis^10^ and the TS repeats are known to bind its targets COMP and thrombospondin-1^7,8^. The authors were able to induce an immune response against these epitopes and subsequently studied the efficacy in vascular diseases^4^. Interestingly, only antibodies against the catalytic domain were efficient in a screening which focused on remodeling after carotid artery ligation as a functional read-out. The resulting vaccine, termed ATS7vac, was then evaluated for efficacy also in wire-injury models of neointima proliferation and models of atherosclerotic plaque formation. Reassuringly, the results from neointima formation secondary to artery ligation were replicated using wire-injury. Moreover, atherosclerotic plaque formation and progression under proatherogenic conditions were reduced by ATS7vac. The effects were impressive and went even beyond the findings of Bauer and colleagues, who studied Apoe-/- mice with genetic deletion of ADAMTS-7 and observed only a borderline reduction of atherosclerotic plaques in the aortic root^9^. An almost 50% reduction of aortic plaques by ATS7vac, however, suggests that targeting ADAMTS-7 in atherosclerosis might be a very promising strategy to address the residual risk of atherosclerosis after controlling cholesterol levels.

In clinical practice, targeting ADAMTS-7 might even be more attractive considering its effects on vascular remodeling. Neointima formation occurs after vascular injury but also after the implantation of coronary stents. In-stent restenosis secondary to the formation of neointima or neoatherosclerosis represent the major complications limiting the use of coronary stents in revascularization procedures as they precipitate recurrent symptoms requiring repeated revascularization and increase mortality^11^. Ma and colleagues investigated the potential of inhibiting ADAMTS-7 to reduce in-stent restenosis using ATS7vac in a swine restenosis model^4^. As suggested by the mouse models, ATS7vac reduced neointima formation after implantation of stents in swine coronary arteries as gauged by histology and optical coherence tomography. The authors do not state which type of stent was used in this model and the effect of ATS7vac on neointima formation might vary for bare metal and drug-eluting stents. The approach, however, seems to be promising in this advanced preclinical model.

How might neutralization of ADAMTS-7 by ATS7vac elicit these beneficial effects? In fact, therapeutic exploration of ADAMTS-7 inhibition would largely benefit from a clear perception of its role in vascular biology and beyond. So far, plausible mechanisms have been reported for ADAMTS-7 in neointima formation^7,8^. In contrast, the downstream mechanisms and targets in atherosclerosis remain incompletely understood, perhaps with the exception of ADAMTS-7-mediated SVEP1 degradation, another ECM protein encoded by a CAD risk gene^12, 13^, also called the sushi, von Willebrand factor type A, EGF and pentraxin domain containing 1 gene. While, in a more general way, the example of ADAMTS-7 reminds us of the “holy grail” of a vaccination against atherosclerosis^2,3^, vaccines against ADAMTS-7 are different from previous anti-atherosclerosis vaccination attempts. ADAMTS-7 was so far not linked to the immune system and although atherosclerotic plaque formation goes hand in hand with vascular inflammation, a direct influence of ADAMTS-7 on leukocyte phenotypes has so far not been shown. Vaccination against ADAMTS-7 in addition opens up a new vista on a class of targets that could potentially not only prevent the disease but also mitigate major complications of its treatment (Fig. 1). It furthermore raises the question whether the findings might be transferable to other ECM proteases. Given that metalloproteases have vital functions in cardiovascular tissue remodeling^14^, this remains questionable. ADAMTS-7 might be unique as it does not seem to be critical in developmental processes given that complete knock-out of ADAMTS-7 in mice did not provoke obvious phenotypes other than arterial protection^8^.

Assuming that the mechanisms affected by vaccination against ADAMTS-7 are better understood, such treatment needs to demonstrate an excellent safety profile, specifically, since novel strategies for prevention of atherosclerosis or treatment of its complications might be of relevance for large patient groups. Importantly, Ma and colleagues observed no safety issues of ATS7vac^4^, which nevertheless needs to be complemented by robust testing in other models before treatment of patients can be considered.

What could be the next steps? Even if ADAMTS-7 inhibition is safe, it will be challenging to prove effects on atherosclerotic plaque formation in primary prevention. In patients who suffered from an acute coronary syndrome, event rates are higher, which might translate to lower patient numbers in clinical trials for assessment of the incidence of cardiovascular events after ADAMTS-7 vaccination. However, even trials in patients with clinical manifestations of atherosclerosis – such as in the CANTOS trial that evaluated the efficacy of the interleukin 1beta-neutralizing antibody canakinumab – require more than 2,000 high-risk patients in each group to detect a ~15% reduction of major cardiovascular events^15^. Although restenosis – apart from stent thrombosis – remains to be the major complication of stent placement, evaluation of novel therapies in humans is challenging since contemporary drug-eluting stent platforms have reduced its incidence to ~10% and hence detection of a clinical benefit might be difficult.

Bearing all these challenges in mind, Ma and colleagues provide a first but very promising step toward a mechanistic diversification of treatment and prevention of atherosclerosis and/or in stent-stenosis (Fig. 1)^4^. Further studies need to corroborate their findings and pave the long way toward the clinical use of a vaccination against ADAMTS-7 in patients at risk for atherosclerosis and its complications.

## Figures and Tables

**Figure 1:**
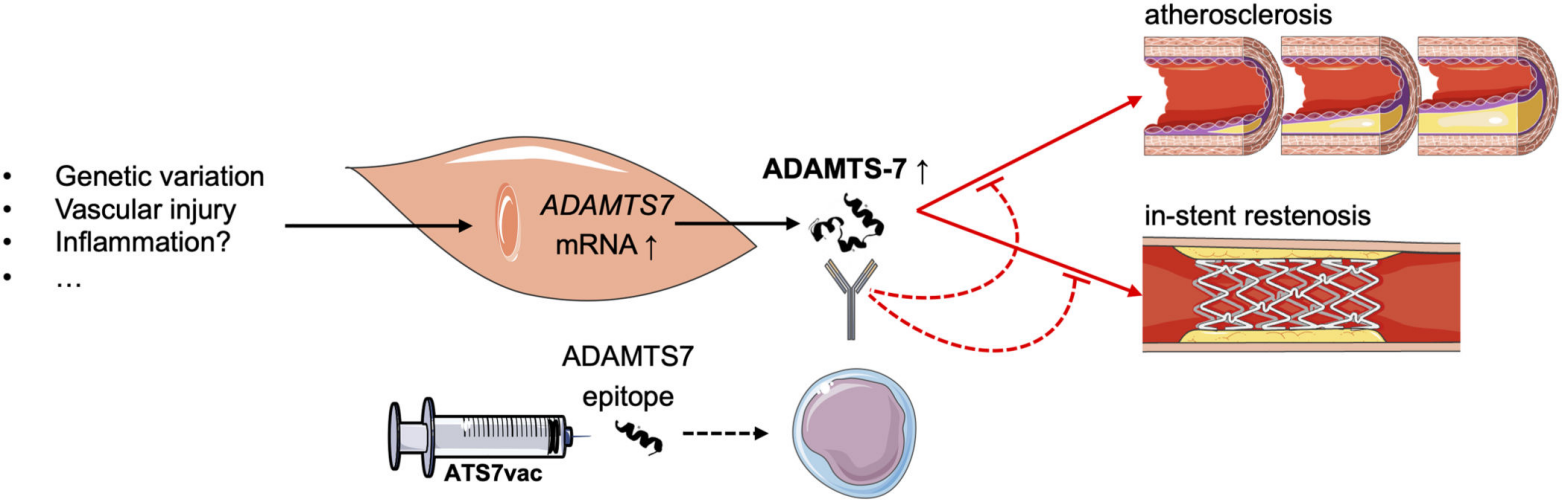
ADAMTS-7 vaccination. ADAMTS-7 expression and activity in vascular smooth muscle cells were found to be increased by a variety of stimuli. ADAMTS-7 modulates atherosclerosis and neointima formation through several known but also unexplored pathways. Vaccination against ADAMTS-7 (ATS7vac) using an ADAMTS-7 epitope leads to the generation of anti-ADAMTS-7 antibodies by B cells. This approach could be used in primary prevention to ameliorate atherosclerotic plaque formation or in patients treated with stents for reducing neointima formation via the inhibition of ADAMTS-7 and its downstream effects.
